# Relevance of Management Science in the One Health Paradigm

**DOI:** 10.1002/hpm.70024

**Published:** 2025-09-23

**Authors:** Federico Cosenz

**Affiliations:** ^1^ Healthcare Management University of Palermo Palermo Italy

**Keywords:** digital transformation, governance, Management Science, One Health, performance management systems

## Abstract

The One Health paradigm recognises the interconnectedness of human, animal, and environmental health, calling for collaborative and cross‐sectoral responses to increasingly complex health threats. However, operationalising this vision requires more than biomedical or ecological knowledge. It requires systems thinking, structured decision‐making, performance evaluation, and governance mechanisms. Management science—including decision analysis, operations research, systems modelling, and digital innovation—can play a pivotal role in transforming the One Health framework from aspirational policy to effective practice. We build upon recent interdisciplinary literature to demonstrate how Management Science is inherently transversal to One Health research fields. Drawing from examples involving artificial intelligence, data governance, and collaborative networks, we propose a revised roadmap for embedding Management Science capacity across One Health platforms, underlining the need for a human‐centred, ethically grounded, and digitally enabled ecosystem for action.

## 1 Introduction

Over the past decade, zoonotic diseases such as Ebola, avian influenza, Covid‐19, and Rift Valley Fever have underscored the fragile interdependencies of human, animal, and ecosystem health. One Health has emerged as the guiding framework for addressing these challenges by fostering interdisciplinary collaboration [1, 2]. Yet, translating this holistic vision into practice often founders on practical hurdles: surveillance systems remain compartmentalised, supply chains break down under stress, and governance arrangements lack mechanisms for shared accountability [3, 4]. These challenges are not merely technical but managerial in nature, suggesting a critical role for Management Science in enabling effective One Health governance. Furthermore, recent work has emphasised the integration of digital tools and artificial intelligence (AI) as key enablers for enhancing One Health analytics and decision‐making [5, 6]. For example, local programs in Sri Lanka have demonstrated how structured frameworks, informed by multi‐criteria decision analysis (MCDA), support sustainable zoonotic disease control strategies tailored to local contexts [7]. Such evidence highlights the urgent need to integrate management tools and capacities at every level of the One Health ecosystem. Management Science, defined as the application of quantitative and organizational methods to complex decision‐making problems, offers proven approaches to overcome these barriers and transform integrative aims into resilient, system‐wide solutions.

## 2 Management Science Methods for One Health Integration

This section illustrates peculiar Management Science methods and provides examples of how they have been applied to highlight insights related to supporting and integrating the three domains of One Health (i.e., human, animal, and environmental health) from a holistic perspective. In particular, given the operational context in which One Health is applied, we refer to the conceptualisation of Health Management provided by a recent scholarly work [8], which defines it ‘*as the practice of providing guidance and leadership to promote and support health at the individual, organisational and systemic levels. Health management embraces a holistic vision of health that reco-gnises the influence of behavioural, social, and environmental determinants*’.

### 2.1 Decision Analysis for Holistic Prioritisation

Decision analysis provides structured frameworks for evaluating interventions against multiple, sometimes competing, objectives. In Southeast Asia, multi‐criteria decision analysis (MCDA) enabled health authorities to compare the relative benefits of rabies vaccination in domestic dogs against wildlife population control and community education [9]. By assigning weights to public health impact, ecological sustainability, and cost, MCDA revealed strategies that simultaneously reduce human morbidity and enhance ecosystem resilience, providing insights unattainable through single‐criterion assessments. The inclusion of stakeholder preferences, environmental impact, and economic cost demonstrates the power of decision science to inform One Health policy and strategy in a holistic and transparent manner [10].

### 2.2 Operations Research in Resource Allocation

Operations research models—including linear and integer programming—offer precision in allocating vaccines, diagnostic kits, and personnel across spatial networks. A study in East Africa employed integer‐programming techniques to redesign Rift Valley Fever vaccine distribution, resulting in a 20% reduction in logistical costs while ensuring coverage of high‐risk herds [11]. These models systematically account for storage capacities, transport times, and risk gradients, providing decision makers with analytically optimised deployment plans rather than anecdotal judgements. In addition, these techniques are particularly useful when supply chains are under stress, as observed during the COVID‐19 pandemic [12].

### 2.3 Systems Modelling and Simulation for Healthcare Performance Management

System dynamics and agent‐based simulations capture feedback loops among human behaviour, animal movements, and environmental drivers, thereby allowing the simulation of disease transmission, ecological changes, and behavioural responses for decision‐support purposes. For example, integrating climate data with predictive migration models of fruit bat populations allowed researchers to forecast potential Ebola spillover hotspots in Central Africa [1]. By simulating the effects of land‐use change scenarios and targeted vaccination campaigns, these virtual laboratories equip policymakers to anticipate outbreaks and test intervention combinations under varying assumptions. The use of digital twin simulations in One Health Living Labs is also emerging as a next step towards real‐time policy testing and performance management [5]. In addition, a study in Tehran, Iran, developed a stochastic, multi‐objective, multi‐period simulation–optimization model for COVID‐19 vaccine supply chains across universities. This model incorporated four echelons—manufacturers, hospitals, vaccination centres, and student volunteers—and simulated dynamic demand fluctuations using system dynamics. By optimising concurrently for cost efficiency, vaccine desirability among students, and equitable distribution, and employing advanced metaheuristic algorithms (Modified Whale Optimization Algorithm combined with Variable Neighbourhood Search), the authors demonstrated how system dynamics modelling can ground vaccine allocation decisions in equitable, cost‐effective, and behaviourally responsive frameworks [13].

### 2.4 Optimising Supply Chain Resilience

Global crises often expose vulnerabilities in supply chains for essential health commodities. Network‐design algorithms can strengthen resilience by identifying critical distribution hubs and alternative routing paths. During the COVID‐19 pandemic, an EU‐wide optimization initiative brought together veterinary, human‐health, and logistics agencies to establish joint procurement and warehousing protocols for personal protective equipment. This cross‐sector task force cut average delivery times by 30% during peak demand periods [12].

### 2.5 Governance Structures and Change Management

Technical solutions require supportive governance to be effective. Research in organizational network governance reveals that clarity of roles, shared performance metrics, and dynamic coordination forums enable disparate actors to form cohesive partnerships [3]. Building such structures involves not only formal agreements but also regular joint exercises, data‐sharing platforms, and investment in collaborative leadership training that spans ministries of health, agriculture, and environment. The success of One Health initiatives often hinges on governance mechanisms, stakeholder trust, and coordination. Scholars, such as [4], have emphasised the fragmentation of One Health networks and the lack of effective monitoring frameworks. Management Science provides the instruments for network governance, performance evaluation, and adaptive leadership development. Creating institutional platforms with shared performance metrics and feedback loops is crucial for advancing system‐wide learning and supporting informed future strategic decisions.

### 2.6 Digital Health, Artificial Intelligence and One Digital Health

Artificial Intelligence (AI) and data platforms are redefining the operational landscape of One Health. From genomic surveillance to real‐time animal health monitoring, AI enables the development of early warning systems and precision public health interventions [6]. A recent research [5] proposes the One Digital Health (ODH) framework, emphasising FAIRER principles—Findable, Accessible, Interoperable, Reusable, Ethical, and Reproducible data—grounded in equitable governance. These approaches call for integration with management tools to ensure both technological feasibility and organizational adoption. Federated learning approaches further allow collaborative model training while preserving data privacy [14]. Ethical AI frameworks emphasise the importance of bias mitigation, transparency, and continuous validation to ensure the responsible adoption of One Health interventions [15].

## 3 Conceptual Framework

Management Science acts as a transversal enabler across One Health research domains. Whether optimising AI‐supported diagnostics in veterinary care, evaluating public health campaigns, or aligning conservation investments, management methods cut across disciplines. The study [2] emphasised the importance of integrating environmental, social, and economic sciences in One Health epidemiology. The intersectoral nature of One Health necessitates approaches that are equally integrative, and this is the role of Management Science.

**FIGURE 1:**
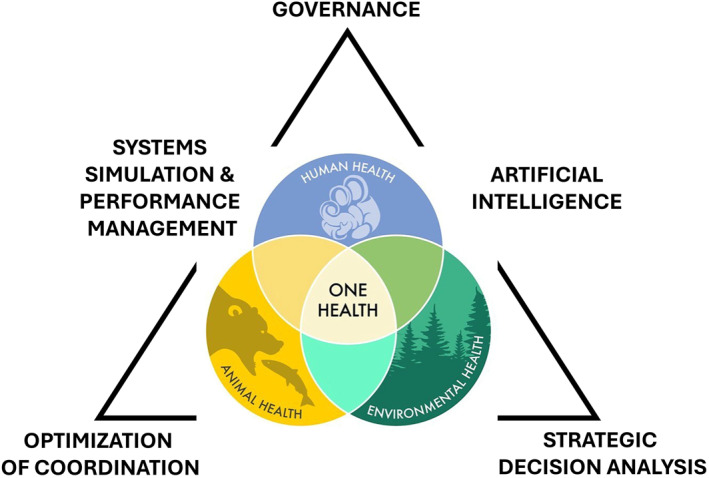
Conceptual model of integration points between Management Science methods and One Health domains.

Figure 1 illustrates the conceptual model of how Management Science methods intersect with the three domains of One Health. Decision analysis tools anchor priority setting across human, animal, and environmental health; optimization algorithms streamline resource flows among these domains; and governance mechanisms provide the connective tissue that sustains collaboration and shared accountability. In the middle, we found systems simulations and digital infrastructures integrating FAIRER data standards and trustworthy AI that provide the backbone for evidence‐driven decisions, ensuring interoperability and inclusivity [16].

While our framework shares the same foundational vision of interconnected health as the WHO Quadripartite Joint Plan of Action and the OHHLEP definition, it differs in emphasis and scope. The WHO Quadripartite Joint Plan of Action is structured around six ‘action tracks’ that identify global priorities—ranging from strengthening capacities to addressing antimicrobial resistance—and provides countries with a theory of change and operational guidance [17]. Similarly, OHHLEP has produced an inclusive definition of One Health and highlighted critical knowledge gaps, particularly the need for intersectoral governance, integrated surveillance, and workforce development [18].

Our proposed framework does not aim to replace or replicate these agendas. Rather, it complements them by introducing Management Science as a transversal enabling layer. Specifically, whereas WHO/OHHLEP frameworks focus on what actions should be taken and why they matter, our framework operationalises how these actions can be prioritised, optimised, coordinated, and evaluated in practice. In doing so, it explicitly embeds decision analysis, operations research, systems simulation, governance design, and digital infrastructures into the One Health paradigm. This shift—from normative policy commitments to structured analytical processes—provides the methodological backbone for transforming high‐level strategic frameworks into actionable, evidence‐informed, and adaptive programs at multiple levels of governance. As such, it fosters the exploration of the interplays across these multiple levels of governance, ranging from macroeconomic, political, and public health down to managerial and organizational echelons, thus promoting interdisciplinary research and practice for a more comprehensive understanding of how to effectively conduct and coordinate One Health initiatives.

## 4 Pathways Forward

To realize the transformative potential of Management Science within One Health, we suggest that three strategic priorities must guide investments and capacity building to enhance the mutual scope and impact of One Health across countries and sectors.

### 4.1 Embed Management Science Units

National and international One Health platforms should establish dedicated units staffed by operations researchers, systems modellers, data scientists, and AI experts. These units would collaborate with epidemiologists, veterinarians, and ecologists to co‐develop context‐specific decision‐support models, calibrate simulations with real‐world data, and facilitate stakeholder workshops that promote a shared understanding of analytical insights. A tangible example is Sciensano's implementation of the national BELMAP report, where researchers conducted a structured stakeholder analysis across human, animal, and environmental health sectors to inform the development of Belgium's One Health antimicrobial use and resistance surveillance [19]. This approach exposed fragmentation in cross‐sector coordination and directly informed governance strategies—illustrating how embedded analytics units can support integrated policy and operational improvements across One Health domains. This also strengthens governance by building adaptive, participatory networks with monitoring frameworks [4].

### 4.2 Invest in Interoperable Data Infrastructures

Sustainable integration depends on data architectures that link human clinical records, animal health surveillance, and environmental monitoring. This involves investing in ethical, inclusive digital infrastructures aligned with FAIRER principles [5]. For example, Canada's Integrated Programme for Antimicrobial Resistance Surveillance (CIPARS) successfully harmonises data across veterinary, human, and retail food sectors, enabling coordinated action against antimicrobial resistance [20]. By contrast, in many low‐ and middle‐income countries, fragmented reporting systems and limited interoperability hinder the use of predictive analytics and rapid response [21]. Addressing these disparities requires standardized ontologies, secure API frameworks, and real‐time performance monitoring dashboards to underpin predictive analytics and rolling‐horizon optimization, empowering decision‐makers to reallocate resources dynamically in response to evolving threats [16].

### 4.3 Pilot Living Labs for Analytics‐Driven One Health

Select regions can serve as One Health Living Labs, where decision‐analysis training, optimization tool deployment, and governance simulations are applied to pressing challenges, such as antimicrobial stewardship across hospital‐farm networks or proactive vaccination strategies in wildlife reservoirs. The *MediLabSecure* initiative, implemented across the Sahel, Middle East, Balkans, and Black Sea regions, offers a precedent. Through joint laboratory capacity‐building, vector surveillance, and simulation exercises, it has demonstrated how regional pilots accelerate knowledge transfer across disciplines, as in the Armenian case [22]. However, evaluations showed that insufficient integration of behavioural and economic modelling hindered policy uptake. To avoid similar pitfalls, future Living Labs should incorporate federated learning and trustworthy AI protocols from the outset, ensuring both equity and privacy. Transparent evaluations of these pilots, coupled with open sharing of successes and challenges, will accelerate system‐wide learning, allowing successful prototypes to be scaled globally.

## 5 Conclusion

The complexity and interdependence of modern health threats demand integrated approaches that go beyond traditional biomedical or ecological expertise [23]. Management Science provides structured, evidence‐based methodologies—ranging from decision analysis and optimization models to systems simulation, digital infrastructures, and governance frameworks—that enable the One Health paradigm to move from an aspirational concept to an actionable and measurable practice.

Future research should aim to strengthen the operationalisation of these methods in several key areas. First, there is a need for longitudinal studies to evaluate the real‐world impact of Management Science tools in diverse One Health contexts, ranging from zoonotic outbreak prevention to environmental risk mitigation. Second, greater emphasis should be placed on developing interoperable digital infrastructures that facilitate real‐time data sharing and advanced analytics, ensuring that FAIRER principles and ethical AI governance guide technological integration. Third, interdisciplinary studies should focus on refining performance simulation models to better capture the complexities of behavioural, social, and environmental factors, thereby enhancing predictive accuracy and supporting informed strategic decisions [24]. Ultimately, participatory research approaches are crucial for co‐creating governance structures that promote trust, equity, and inclusivity, thereby ensuring that One Health strategies are culturally relevant and globally scalable.

By fostering collaborative innovation and investing in adaptive research agendas, the One Health community can leverage Management Science to enhance preparedness, optimise resource allocation and related outcomes, and create resilient, digitally enabled health ecosystems for humans, animals, and the environment alike.

## Conflicts of Interest

The author declares no conflicts of interest.

## Data Availability

Data sharing not applicable to this article as no datasets were generated or analysed during the current study.
